# Dewetting of monolayer water and isopropanol between MoS_2_ nanosheets

**DOI:** 10.1038/s41598-018-35163-3

**Published:** 2018-11-12

**Authors:** Beibei Wang, Rajiv K. Kalia, Aiichiro Nakano, Priya D. Vashishta

**Affiliations:** 1 Collaboratory of Advanced Computing and Simulations, University of Southern California, Los Angeles, USA; 2 Department of Physics and Astronomy, University of Southern California, Los Angeles, USA; 3 Mork Family Department of Chemical Engineering and Materials Science, University of Southern California, Los Angeles, USA; 4 Department of Computer Science, University of Southern California, Los Angeles, USA

## Abstract

Understanding dewetting of solvent molecules confined to layered material (LM) interfaces is crucial to the synthesis of two-dimensional materials by liquid-phase exfoliation. Here, we examine dewetting behavior of water and isopropanol/water (IPA/H_2_O) mixtures between molybdenum disulfide (MoS_2_) membranes using molecular dynamics (MD) simulations. We find that a monolayer of water spontaneously ruptures into nanodroplets surrounded by dry regions. The average speed of receding dry patches is close to the speed of sound in air. In contrast, monolayer mixtures of IPA/H_2_O between MoS_2_ membranes slowly transform into percolating networks of nanoislands and nanochannels in which water molecules diffuse inside and IPA molecules stay at the periphery of islands and channels. These contrasting behaviors may explain why IPA/H_2_O mixtures are much more effective than H_2_O alone in weakening interlayer coupling and exfoliating MoS_2_ into atomically thin sheets.

## Introduction

Understanding the behavior of liquid films at solid interfaces is important in a variety of applications such as self-removal of liquids, self-cleaning, antifogging^1–6^, self-assembly^7^ of microscopic clusters, and water harvesting technology^8^ in which liquid molecules adsorbed on a solid surface aggregate and condense into droplets through dewetting. Strong interaction between adsorbed liquid and solid surfaces affects and modulates many interfacial processes, including but not limited to molecular transport^9^, surface corrosion^10^, and chemical reactivity^11,12^. Motivated by these applications, numerous studies of the dynamics of liquid-film dewetting have been carried out. Mulji and Chandra^13^ have studied the rupture and dewetting of thin water layers on solid substrates with different surface conditions. Their results explain how the rupture of a water film starts from the boundary of a hydrophobic or hydrophilic surface. Jensen *et al*.^14^ have examined the role of interfacial energy in dewetting of water on a hydrophobic surface.

The behavior of liquids confined to solid surfaces has been studied extensively with molecular dynamics (MD) simulations. Zhang *et al*.’s studies^15^ of water nanofilms in contact with silica surfaces indicate that dewetting is a two-step process in which dry patches appear due to thermal fluctuations and water film contracts because of hydrogen bonding and electrostatic interactions. Kayal and Chandra^16^ performed MD simulations to study dewetting of water in carbon nanotubes. They found that dewetting, tunnel flow, and molecular orientation of H_2_O could be tuned by an electric field applied normal to the direction of H_2_O transport.

In this paper, we report MD simulation studies of dewetting of isopropanol and water (IPA/H_2_O) mixtures confined between molecularly thin MoS_2_ membranes. Understanding the structure and dynamics of solvent molecules between transition metal dichalcogenide (TMD) layers is key to the synthesis of two-dimensional layered materials (LMs) by liquid-phase exfoliation (LPE) using ultrasonication or shear. Despite a great deal of experimental work on LPE of TMDs^17–23^, there is very little understanding of structural characteristics and dynamics of solvent molecules in the galleries of TMDs or how solvent molecules weaken the interaction between TMD layers to cause exfoliation into atomically thin sheets.

Our MD simulations reveal distinct dewetting processes for water and IPA/H_2_O mixtures in the galleries of MoS_2_ bilayers. In the case of water, we find that the contact line separating dry and wet patches recedes at the speed of sound waves in air to cause spontaneous break-up of the H_2_O film into nanodroplets and, concurrently, MoS_2_ deforms to accommodate these nanodroplets. In contrast, the speed of the receding contact line is noticeably reduced in the presence of IPA molecules because of their low diffusivity. An IPA/H_2_O film spontaneously transforms into a percolating network of islands connected by narrow channels in which H_2_O molecules are mostly inside and IPA molecules are at the periphery of those islands and channels.

## Method

In the simulations reported here, reactive empirical bond order (REBO)^24^ potential is used to describe the interaction between Mo and S atoms in MoS_2_ and TIP4P/2005^25^ force field is used for H_2_O molecules. The MoS_2_ REBO potential accounts for changes in local atomic configurations of atoms and TIP4P/2005 correctly models structural and dynamical properties of bulk and nanoconfined water. The interaction between IPA molecules is described by OPLS-AA^26^ force field, which is commonly used for a variety of organic molecules. The interaction between MoS_2_ membranes and water^27^ is modeled by Lennard-Jones (L-J) potentials between Mo-O and S-O pairs, and interactions between Mo-O, Mo-C, S-O, and S-C pairs in IPA and MoS_2_ are also described by L-J potentials. The force fields for MoS_2_, H_2_O and IPA include long-range Coulomb potential between all charged particles. By modeling MoS_2_-solvent interface^28^, we optimize the force-field with experimental data on contact angles for H_2_O and IPA/H_2_O droplets on an MoS_2_ substrate. The force-field parameters are listed in Table S1 and the procedure is described in the supplementary material.

The simulation setup is shown in Fig. S3 of supplementary material. Initially, the system consists of a monolayer of IPA/H_2_O mixture between two atomically-thin MoS_2_ membranes of dimensions 100 nm × 100 nm. Water and IPA molecules are distributed randomly at a height of about 3 Å above an MoS_2_ [001] surface and the second MoS_2_ sheet is placed on top of the solvent at a distance of 3 Å from liquid molecules. The IPA concentration is 50% by weight. Periodic boundary conditions are applied parallel to the membranes, i.e., along *x* and *y* directions, and equations of motion for atoms are integrated with the Velocity-Verlet algorithm using a time step of 1 femtosecond.

## Results

**Figure 1:**
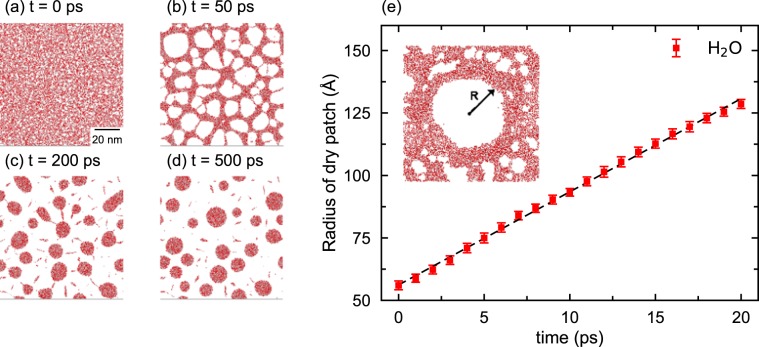
Dewetting of an H_2_O film between MoS_2_ membranes. Red regions represent H_2_O. (**a**) Top view of the H_2_O monolayer at *t* = 0 ps. Dry patches appear spontaneously and start expanding rapidly. (**b**) H_2_O molecules form a network structure at *t* = 50 ps. (**c**) At *t* = 200 ps, the network breaks up into H_2_O droplets. Some of them coalesce to form larger droplets. (**d**) H_2_O dewetting at *t* = 500 ps. (**e**) Radius of a dry hole in the film expands linearly with time and the slope gives a dewetting speed of 373 m/s.

Let us first examine dewetting in the reference system consisting of a water monolayer between a pair of MoS_2_ membranes. Figure 1(a) shows the initial configuration; (b), (c) and (d) are three snapshots show the break-up of the monolayer into nanodroplets (red) and small dry patches (white). (MoS_2_ membranes are not displayed here for the sake of clarity). At the onset of dewetting, we observe small dry patches at random locations in the film. They appear due to thermal fluctuations on the picosecond time scale. After 50 ps, the film breaks up into a network of H_2_O nanodroplets connected by thin H_2_O channels. The latter disappear after 200 ps, leaving behind isolated nanodroplets and a much larger fraction of dry patches. The snapshot in Fig. 1(d) shows the result of Rayleigh instability causing the break-up of the monolayer into nanodroplets of diameters ranging between 5 and 20 nm and a few isolated short chains of H_2_O molecules. The whole process of rupturing of the liquid film takes only 500 ps.

Theoretically, it is known that the contact line, i.e., the edge of a dry patch, recedes at a constant speed under specific conditions. To examine whether it is true for dewetting of water between MoS_2_ membranes, we performed another simulation in which the initial configuration had a dry circular hole of radius 5 nm at the center of the monolayer (see the insert in Fig. 1(e)). The initial dry patch was created by removing water molecules in the middle of the monolayer. We monitored the growth of the dry patch as a function of time by tracking the distance between several pairs of points on opposite sides of the dry patch. Figure 1(e) shows that the average separation between those pairs of points on the circular patch increases linearly with time. The speed of the dry patch growth, i.e. the dewetting velocity, estimated from the slope is ~373 m/s. This is close to the speed of sound wave in air at room temperature. However, the dewetting velocity estimated from Culick’s law ($v={(\frac{2{\gamma }_{sl}}{\rho e})}^{(1/2)}$, where the numerator is the interface energy and the denominator, which is the product of density *ρ* and thickness *e*, gives the surface density of the liquid) is 467 m/s. The discrepancy from our MD result arises from the fact that Culick’s law is based solely on the conversion of interface energy into kinetic energy of dewetting and does not take into account the deformation of MoS_2_ membranes which we observe during the dewetting process (see Fig. S4 in supplementary material).

**Figure 2:**
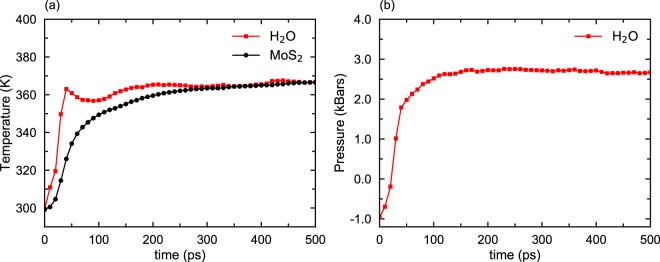
Temperature and pressure of the system during dewetting. (**a**) “Temperature” of water rises quickly in the first 50 ps of dewetting. Subsequently, the energy transferred from water to MoS_2_ increases the temperature of MoS_2_ membranes. The equilibrium is reached after 300 ps. (**b**) Shows that pressure increases rapidly as the water monolayer breaks up into nanodroplets.

Our simulation also reveals that rapid dewetting of water is accompanied by significant increases in the temperature and pressure of the entire system. Figure 2(a) shows that the “temperature” (a measure of instantaneous kinetic energy) of water increases from 300 K to 360 K within 40 ps while the same temperature increase in MoS_2_ takes place in 250 ps. Water nanodroplets and MoS_2_ membranes come to an equilibrium after 300 ps and the temperature of the system remains at 360 K.

Figure 2(b) shows that pressure in water due to the deformation increases to 2,700 bars in the first 100 ps of dewetting but does not change subsequently. This increase in pressure does not change the density of water nanodroplet, but produces ripples in MoS_2_ membranes, and dimples are formed on the membranes after dewetting (see Fig. S4 in supplementary material). A small fraction of water molecules forms a triangular structure in registry with the MoS_2_ lattice (Fig. S5 in supplementary material). The contact angle of water droplets is around 25°, which is much less than the contact angle of a standalone H_2_O droplet on an MoS_2_ substrate (97°).

**Figure 3:**
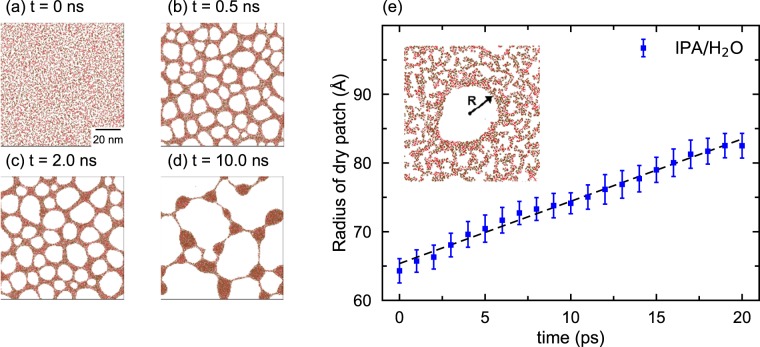
Dewetting of an IPA/H_2_O mixture between MoS_2_ membranes. (**a**) Snapshot of the IPA/H_2_O liquid film *t* = 0 ps. (**b**) Snapshot of the mixture taken at *t* = 0.5 ns shows a percolation network in which IPA and H_2_O molecules have phase separated. IPA molecules are mostly outside and H_2_O are inside the network. (**c**) Snapshot of the IPA/H_2_O mixture *t* = 2.0 ns shows an increase in the fraction of dry patches. (**d**) Shows the formation of interconnected nanodroplets after 10 ns. (**e**) Shows a linear increase in the radius of a circular dry patch (inset). The speed of the dry patch in the mixture is significantly less than that in pure water.

The behavior of IPA/H_2_O mixtures is significantly different from the dewetting of puer H_2_O. Figure 3(a–d) are MD snapshots of a mixture consisting of 50% IPA by weight between MoS_2_ membranes. Figure 3(a) shows the initial configuration of the system. Thermal fluctuations begin to create small tears in the film. Figure 3(b) indicates that ruptures have grown into relatively large dry patches (white) after 0.5 ns, and these patches keep on expanding with time as shown in Fig. 3(c,d). Liquid nanodroplets appear between the membranes after 10 ns. They are connected by narrow channels of the mixture, see the snapshot in Fig. 3(d). We also notice that the attraction between H_2_O and IPA causes water molecules to aggregate around bigger IPA molecules instead of being randomly distributed over the entire wet region.

We have also examined the time evolution of a dry circular patch in an IPA/H_2_O monolayer. Figure 3(e) shows that the average radius of the dry circular patch increases almost linearly with time, albeit much more slowly than the expansion of a dry patch in an H_2_O film. The slope of the straight line in Fig. 3(e) gives an estimate of the dewetting velocity to be around 91 m/s, which is significantly slower than the dewetting velocity in the H_2_O film. This is due to the fact that IPA molecules are much bigger and hence diffuse much more slowly than H_2_O molecules.

**Figure 4:**
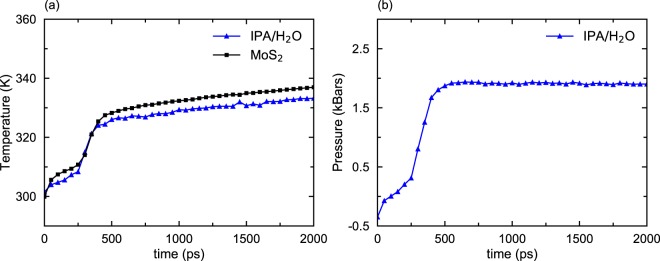
Temperature and pressure of the IPA/H_2_O film during dewetting. The temperature (**a**) and pressure (**b**) of IPO/H_2_O change rapidly in the first 500 ps. Subsequently, the temperature of the mixture and MoS_2_ membranes change slowly and thermal equilibrium is not established even after 3 ns. In contrast, the equilibrium is reached within 200 ps in the absence of IPA.

We have also monitored the temperature and pressure of IPA/H_2_O mixtures during dewetting. Figure 4(a) shows how the temperature of an IPA/H_2_O mixture (1:1 ratio by weight) changes with time. The temperature of the solvent is slightly higher than the temperature of the membranes, indicating that they have not reached equilibrium even after 2 ns. Also note that the temperature increases in the mixture and MoS_2_ membranes are much less than in the case of pure water. Figure 4(b) shows that the pressure in the mixture due to the deformation of MoS_2_ membranes also increases much more slowly than in the case of water. The pressure goes up to 1900 bars, which is 30% smaller than the pressure in the previously mentioned water case, indicating a weaker intra-layer coupling.

**Figure 5:**
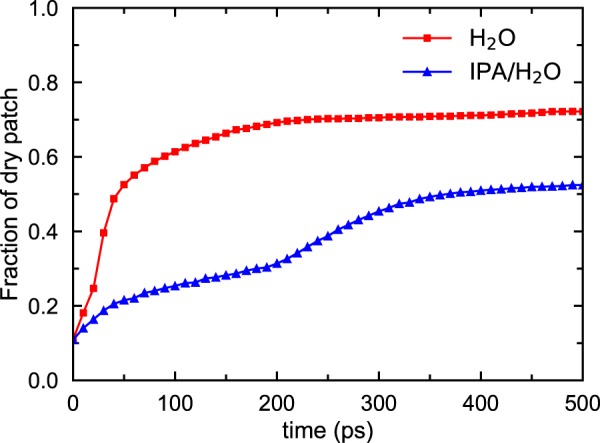
Time evolution of dry patches in H_2_O and 50% IPA/H_2_O mixture during dewetting between MoS_2_ membranes. Dewetting is much slower in the mixture case because of the slow diffusivity of IPA molecules. The fraction of dry patch in the mixture is lower than that of pure H_2_O, indicating that the mixture covers more surface area of MoS_2_.

Another apparent difference between the dewetting of H_2_O and IPA/H_2_O monolayers is in the growth rates of dry patches. Figure 5 shows the fraction of total dry areas for pure H_2_O and an IPA/H_2_O mixture as a function of time. The fraction of the dry patch in IPA/H_2_O is lower because wet patches are connected by ligaments in the network structure. The growth rate in H_2_O dewetting plateaus around 200 ps, whereas dry patches continue to increase slowly during dewetting in the IPA/H_2_O mixture.

In many sonication experiments, the organic solvent for MoS_2_ exfoliation is an IPA/H_2_O mixture^18,22,23,29^. During exfoliation, shear stress or sonication shockwave increases the separation between MoS_2_ layers, allowing solvent molecules to enter the galleries of MoS_2_. Our MD simulations explain why the IPA/H_2_O mixture is more effective than pure H_2_O as a solvent in the exfoliation of MoS_2_. The mixture is spread out over the membranes in the form of a network of islands connected by channels and this spreading of the mixture weakens the interlayer attraction between MoS_2_ bilayer. Water monolayer, on the other hand, breaks up into nanodroplets very quickly, and therefore is not as effective in weakening the interlayer coupling in MoS_2_. These contrasting dewetting processes shed light on the effectiveness of IPA/H_2_O mixture in exfoliation of MoS_2_.

## Conclusion

In conclusion, MD simulations reveal distinct dewetting processes in H_2_O and IPA/H_2_O monolayers confined between MoS_2_ bilayers. An H_2_O monolayer spontaneously ruptures into wet and dry patches with a high velocity (373 m/s) and wet patches agglomerate to form nanodroplets, which cause local deformations in MoS_2_ membranes. The entire dewetting process takes about 500 ps, and it is accompanied by significant increases in temperature and pressure of both H_2_O and MoS_2_. In contrast, the breakup of an IPA/H_2_O monolayer is much slower and temperature and pressure increases are much less than those in the dewetting of a water monolayer. The velocity of dry patches is 91 m/s, and it takes nearly 10 ns before an IPA/H_2_O film finally transforms into a percolating network of islands connected by narrow channels in which H_2_O molecules diffuse inside and IPA molecules at the peripheries of the network. The surface area covered by the percolating network of IPA/H_2_O is much larger than the area covered by H_2_O nanodroplets. This may explain why mixtures of H_2_O and IPA are more effective than pure water in liquid phase exfoliation of MoS_2_^22^.

In addition to IPA/H_2_O mixture we have examined wetting-dewetting transition in a monolayer of Methanol/Water (MeOH/H_2_O) mixture (50% by weight) sandwiched between a bilayer of MoS_2_. This is solvent is also commonly used in sonication exfoliation of MoS_2_. The dewetting phenomenon in this case is very similar to that of IPA/H_2_O. A monolayer of MeOH/H_2_O solvent breaks up into nanodroplets linked by liquid nanochannels, and water molecules reside mostly inside and MeOH at the periphery of nanodroplets and nanochannels.

## Electronic supplementary material

supplementary information
