# Supplementary material for: Dewetting of monolayer water and isopropanol between MoS2 nanosheets
Source: Sci Rep. 2018 Nov 12;8:16704. doi: 10.1038/s41598-018-35163-3 (PMC6232136; doi:10.1038/s41598-018-35163-3)
Supplement: Supplementary file 1 — supplementary information [file 41598_2018_35163_MOESM1_ESM.docx]

# **Supplementary Information**

# **Dewetting of monolayer water and isopropanol between MoS_2_ nanosheets**

Beibei Wang^1,2^, Rajiv K. Kalia^1,2,3^, Aiichiro Nakano^1,3^, Priya D. Vashishta^1,3^

Collaboratory of Advanced Computing and Simulations^1^, University of Southern California, university park campus, Los Angeles, CA 90089.

Department of Physics and Astronomy^2^, University of Southern California, university park campus, Los Angeles, CA 90089.

Mork Family Department of Chemical Engineering and Materials Science^3^, University of Southern California, university park campus, Los Angeles, CA 90089.

*Corresponding Author:

Phone: +1-213-821-2663, +1-213-245-7788

FAX: +1- 213-821-2664

E-mail: [rkalia@usc.edu](mailto:rkalia@usc.edu), [beibeiw@usc.edu](mailto:beibeiw@usc.edu)

Homepage: <https://magics.usc.edu/>

**Interaction parameters:**

The force fields for MoS_2_, H_2_O and IPA include long-range Coulomb potential between all charged particles. Force field parameters for H_2_O can be found in reference **25**. The interaction parameters between MoS_2_ and H_2_O molecules listed in tables S1 can be found in reference **19**. The force field between IPA/H_2_O and MoS_2_ are optimized with experimental values of contact angles for IPA/H_2_O droplets on an MoS_2_ substrate, which is developed in our group and will be published soon.

Table S1. Parameters for Coulomb and L-J interactions between H_2_O and MoS_2_

| Coulomb Interaction | | | | | |
| --- | --- | --- | --- | --- | --- |
| Atoms | Mo | | S | O(H_2_O) | H(H_2_O) |
| Charge (e) | 1.2 | | -0.6 | -1.1128 | 0.5564 |
| L-J Interaction | | | | | |
| Atom Pair | | Mo-O | S-O | Mo-H | S-H |
| $\epsilon$*_ij_* (eV) | | 4.996E-3 | 6.929E-3 | 0.000 | 0.000 |
| $\sigma$*_ij_* (Å) | | 2.869 | 3.344 | 0.000 | 0.000 |

We performed DFT calculation for the coupling strength between two MoS_2_ layers and compared with the MD result based on the REBO forcefield. The DFT cohesive energy is 18.6 meV/Å^2^ for an interlayer separation of 2.9 Å, which is the equilibrium separation in our MD simulation. The DFT calculation is based on Perdew’s recent vdW functional SCAN+rVV10, which is good at reproducing interlayer non-bonded interactions^1^. The MD result for the cohesive energy is 11.2 meV/ Å^2^.

**Contact Angle:**

We calculated the interface energy between the solvent and MoS_2_ from contact-angle simulation and compared the results with experiment. The macroscopic contact angle is estimated using Young’s equation:

$$\cos\left( \boldsymbol{\theta} \right) \boldsymbol{=}\cos\left( \boldsymbol{\theta}_{\boldsymbol{\infty}} \right)\boldsymbol{-}\frac{\boldsymbol{\tau}}{\boldsymbol{\gamma}_{\boldsymbol{v}}\boldsymbol{R}}$$

where $\boldsymbol{\tau}$ is the free energy correction, $\boldsymbol{\gamma}_{\boldsymbol{v}}$ is the surface tension of the solvent, and R is the radius of the contact area (see reference 27 in the paper).

To set up the contact angle simulation, MoS_2_ and the solvent are equilibrated separately for 5 nanoseconds. Subsequently, nanodroplets of different sizes are placed on the MoS_2_ surface at a height of 3 Å. Figure S1 shows the set up for the contact-angle simulation of H_2_O nanodroplets. After 2 nanoseconds of relaxation, the system reaches thermal equilibrium and the density distribution is analyzed in cylindrical coordinates. Figure S2 shows the density profile of a H_2_O droplet. After taking into account the size effect, we find that the contact angle of H_2_O droplet on MoS_2_ surface is 98.9°. The experimental value is 97.8°.

|  |  |
| --- | --- |
| 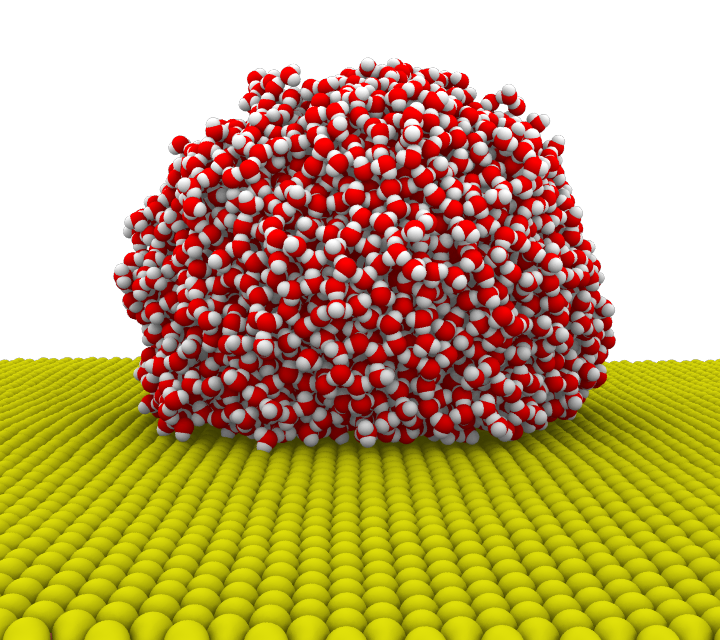 | |
|  | |
| **Figure S1: MD simulation schematic set up for contact angle simulation.** O and H atoms are red and white, respectively; S atoms are yellow, and Mo atoms are not shown in here | |

|  |  |
| --- | --- |
| 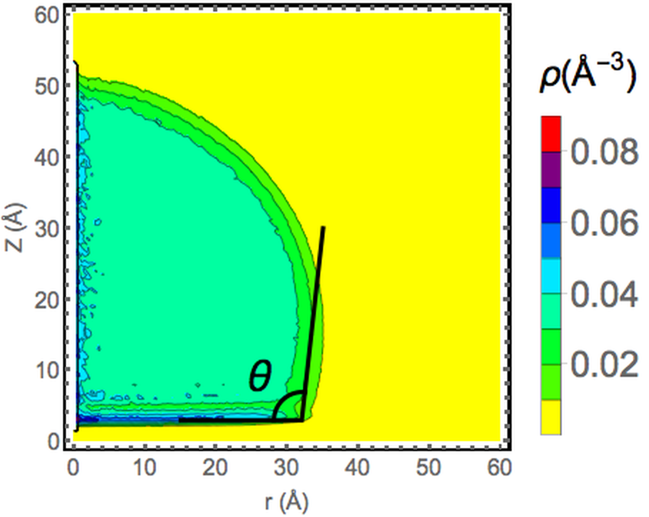 | 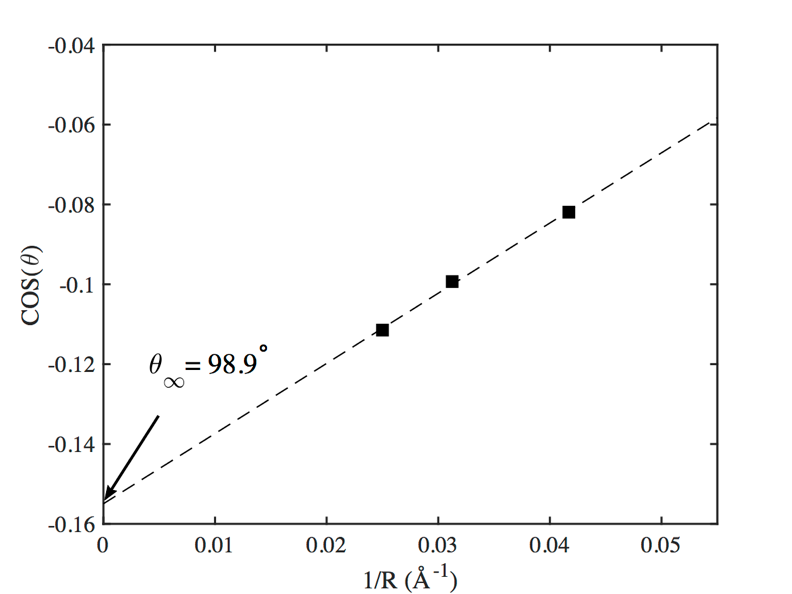 |
| (a) | (b) |
| **Figure S2: Density profile of a H_2_O droplet and the contact angle as a function of droplet size.** (a) schematic diagram of the contact-angle measurement. The colored map represent the density distribution in cylindrical coordinates. Z is the height from MoS_2_ substrate, and r is the distance from the central axis of symmetry of the droplet. $\theta$ is the contact angle at the interface of H_2_O and MoS_2_. (b) contact angle as a function of radius of the contact area. | |

**Dewetting simulation setup and results:**

The following supplementary figures concern the structure of MoS_2_ nanosheets and the liquid nanodroplets trapped inside the nanosheets.

|  |  |
| --- | --- |
| 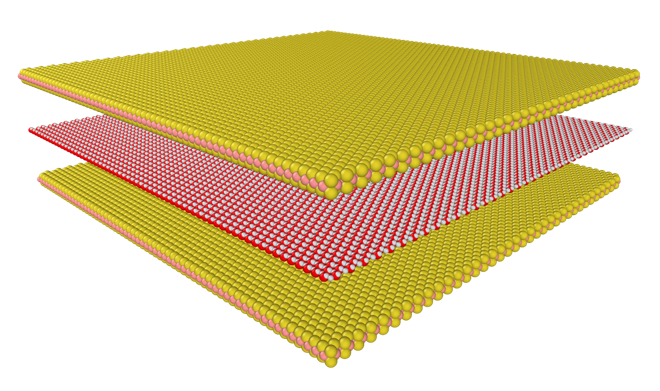 | 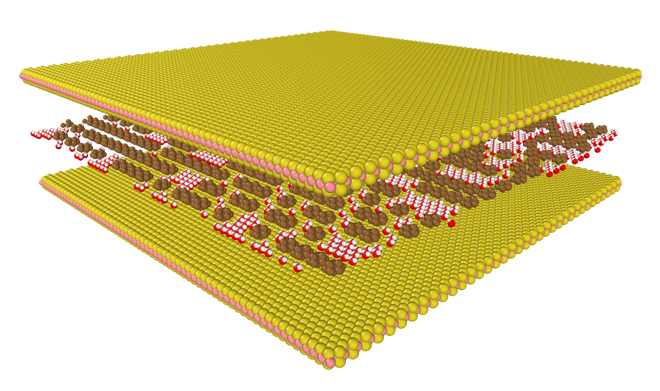 |
| (a) | (b) |
| **Figure S3: MD simulation set ups for (a) H_2_O monolayer, and (b) IPA/H_2_O mixture between MoS_2_ bilayers**. For visualization purposes, MoS_2_ layers are lifted to expose the liquid layer. Oxygen and hydrogen atoms are red and white, respectively; Mo and S atoms are pink and yellow, respectively, and IPA molecules are brown. Periodic boundary conditions are applied in the planes (*x* and *y* directions) of MoS_2_ bilayers, and there are vacuum layers above and below the system so that MoS_2_ layers do not interact with their images in the *z* direction. The size of the system is about 100nm × 100nm in the *x* and *y* directions. | |

|  |  |
| --- | --- |
| 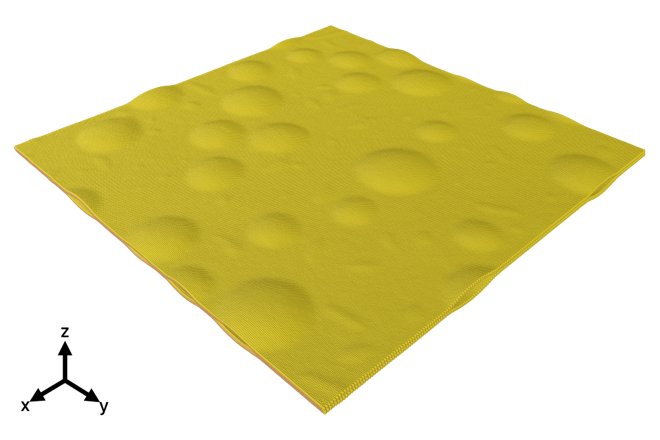 | 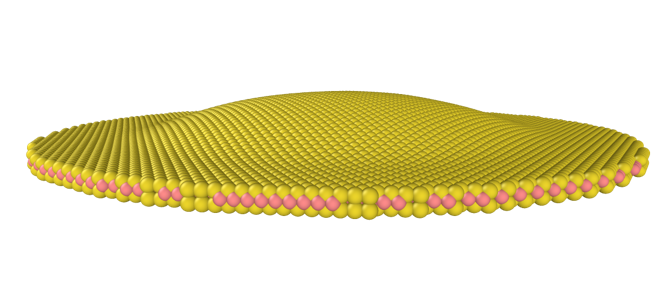 |
|  | (b) |
|  | 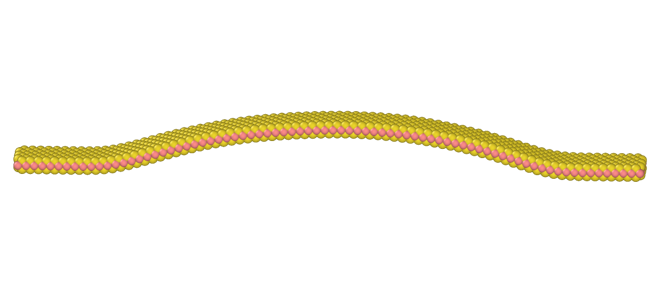 |
| (a) | (c) |
| **Figure S4: Deformation of MoS_2_ membranes caused by dewetting of the H_2_O film**. (a) Dimples are formed in MoS_2_ membranes after dewetting. (b) Close-up view of deformation in MoS_2_. (c) Shows a strip cut from the deformed MoS_2_ cap. Knowing the bending energy and bending force on the strip, we calculate the bending modulus to be 100$\pm$20 N/m. | |

The coupling strength in the presence of H_2_O is 7.1 meV/Å^2^, which is 36.6% smaller than that without H_2_O.

|  |  |
| --- | --- |
| 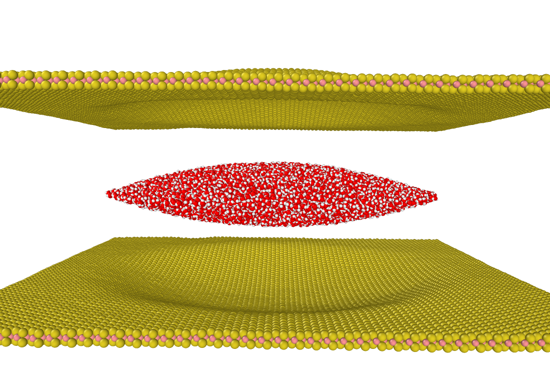 |  |
| (a) | (b) |
| 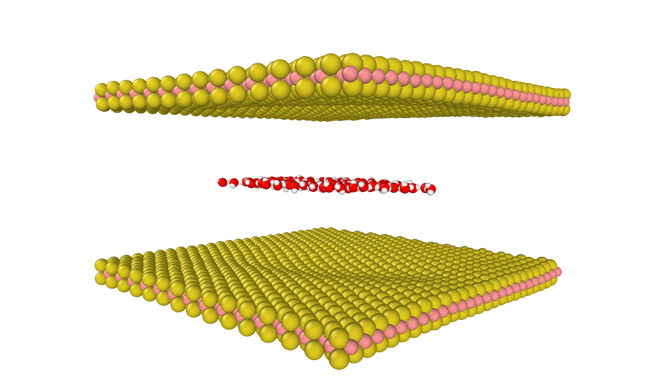 | 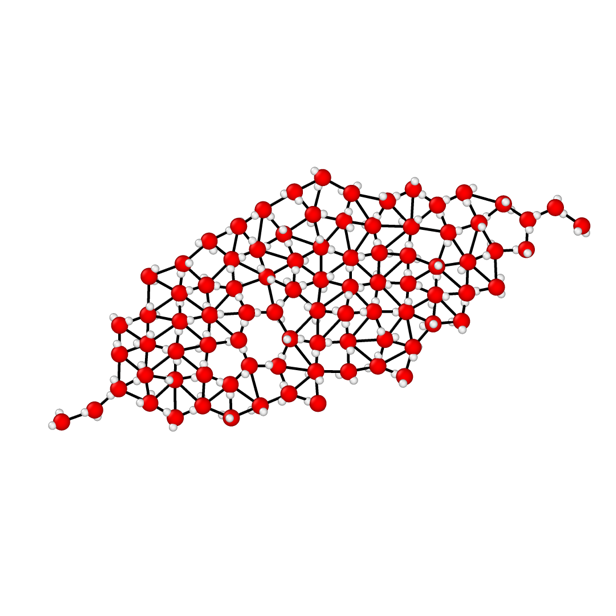 |
| (c) | (d) |
| **Figure S5: Water molecules trapped between MoS_2_ membranes form two types of structures**. (MoS_2_ membranes are lifted to visualize the structures of water.) (a) Water nanodroplet confined inside an MoS_2_ cage. (b) Oxygen-Oxygen radial distribution function of H_2_O in the confined nanodroplet. (c) Confined water molecules form a monolayer of “frozen” H_2_O in registry with the MoS_2_ lattice, and (d) shows the triangular lattice structure formed by water molecules. | |

|  |  |
| --- | --- |
| 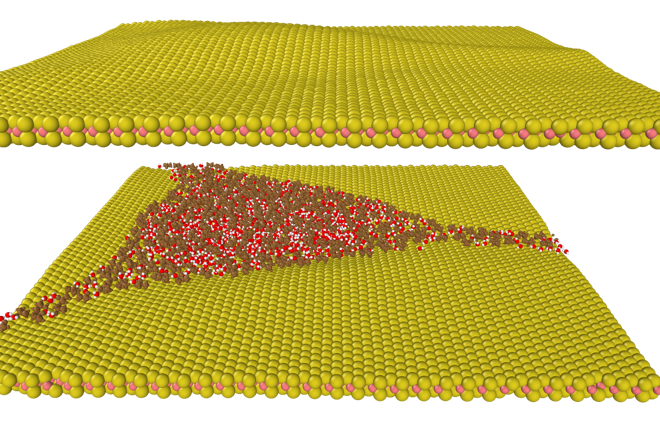 | 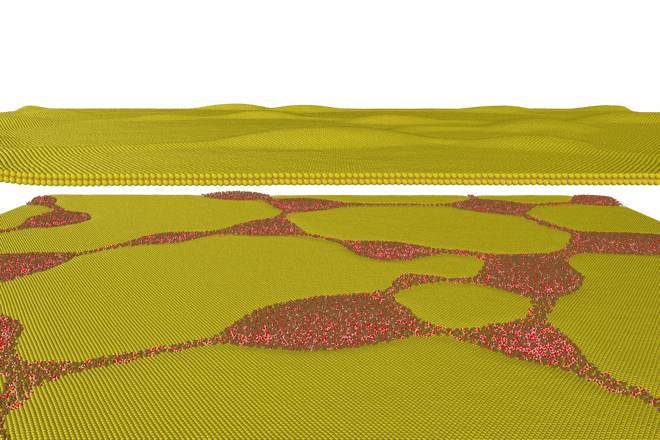 |
| (a) | (b) |
| **Figure S6:** Shows that IPA/H_2_O mixture does not form standalone nanodroplets even after 10 ns. Instead, the droplets remain connected by nano channels. (a) Shows a nanodroplet in which IPA molecules are at the periphery and water molecules are inside the droplet. (b) Shows liquid nano channels connecting nanodroplet islands. MoS_2_ is deformed as shown in (a) and (b). | |

The cohesive energy of MoS_2_ bilayer in the presence of IPA/H_2_O mixture is 4.6 meV/Å^2^, which is 34.7% smaller than that of MoS_2_ with pure H_2_O. We also performed simulations involving sub-monolayer and two layers of IPA/H_2_O mixture between MoS_2_ sheets. In the sub-monolayer case, we again observe the formation of nanodroplets linked by nanochannels during the wetting-dewetting transition. In the case of two layers of the solvent, we ran the simulation for 5 ns but did not observe the dewetting phenomenon. This implies that the second layer of the solvent is highly effective in the exfoliation of MoS_2_.

References:

1. Peng, H., Yang, Z.-H., Sun, J. & Perdew, J. P. SCAN+rVV10: A promising van der Waals density functional. *Phys. Rev. X,* **041005,** 1–15 (2015).
